# Prognostic value of systemic immune-inflammation index in patients with urologic cancers: a meta-analysis

**DOI:** 10.1186/s12935-020-01590-4

**Published:** 2020-10-12

**Authors:** Yilong Huang, Yunfeng Gao, Yushen Wu, Huapeng Lin

**Affiliations:** 1 Department of Radiology, The First People’s Hospital of Chongqing Liang Jiang New Area, Chongqing, China; 2 Department of Urology Surgery, The First Affiliated Hospital of Chongqing Medical University, Chongqing, China; 3 Department of Urology Surgery, The Second People’s Hospital of Chengdu, Chengdu, Sichuan China; 4 Chongqing Key Laboratory of Molecular Oncology and Epigenetics, The First Affiliated Hospital of Chongqing Medical University, 1 Youyi Road, Yuzhong District, Chongqing, 400042 People’s Republic of China; 5 Department of Intensive Care Unit, Affiliated Hangzhou First People’s Hospital, Zhejiang University School of Medicine, 261 Huansha Road, Hangzhou, 310006 Zhejiang People’s Republic of China

**Keywords:** Urologic cancer, Meta-analysis, Prognosis, Systemic immune-inflammation index (SII)

## Abstract

**Background:**

Several studies have reported that the systemic immune-inflammation index (SII) is associated with the prognosis of patients with urologic cancers (UCs). The aim of this study was to systematically evaluate the prognostic value of SII in UC patients.

**Methods:**

We searched public databases for relevant published studies on the prognostic value of SII in UC patients. Hazard ratios (HRs) and 95% confidence intervals (CIs) were extracted and pooled to assess the relationships between SII and overall survival (OS), progression-free survival (PFS), cancer-specific survival (CSS), overall response rate (ORR) and disease control rate (DCR).

**Results:**

A total of 14 studies with 3074 patients were included. From the pooled results, we found that high SII was associated with worse overall survival (OS) in patients with UC (HR 2.58, 95% CI 1.59–4.21). Patients with high SII values also had poorer PFS (HR 1.92, 95% CI 1.29–2.88) and CSS (HR 2.58, 95% CI 1.36–4.91) as well as lower ORRs (HR 0.40, 95% CI 0.22–0.71) than patients with low SII values. In addition, the subgroup analysis of OS and PFS showed that the prognosis of patients with high SII was worse than that of patients with low SII.

**Conclusions:**

SII might be a promising noninvasive predictor in patients with UC. However, more samples and multicenter studies are needed to confirm the effectiveness of SII in predicting the prognosis of patients with UC.

## Background

Urologic cancer is a group of cancers that occur in the urinary system. The incidence of urologic cancer is still high. Kidney cancer is the seventh most common malignancy in men and the ninth most common malignancy in women globally [1]. Bladder cancer is the fourth and eleventh most common cancer among men and women worldwide [2]. Prostate cancer is the most common type of cancer in men and the second leading cause of cancer-related death in men [2]. Despite advances in the early diagnosis and treatment of urologic cancers, the prognosis remains poor due to local recurrence or distal metastasis [3, 4]. Therefore, noninvasive detection tools such as serum biomarkers are increasingly valued for their simplicity and predictive value.

Inflammation is an important predictor of tumor invasion, progression and metastasis [5]. Therefore, a series of biological indicators based on inflammation and/or nutritional status, such as the neutrophil-to-lymphocyte ratio (NLR) and platelet-to-lymphocyte ratio (PLR), have been reported as efficient tumor biomarkers [6–8]. The systemic immune-inflammation index (SII), as a relatively new inflammatory index based on peripheral lymphocyte, neutrophil, and platelet counts, was evaluated to have high diagnostic value for the prognosis of cancer [9, 10]. Poor outcomes have been recently reported in patients with high SII values based on studies of other cancers, such as respiratory system cancers and digestive system cancers [7, 8, 11]. There is still a debate for the use of SII in urologic cancers, although an increasing number of studies has been performed on this topic, and the sample size in the existing research is not that large [12, 13]. Therefore, we conducted a meta-analysis to investigate the prognostic role of SII in patients with urologic cancers.

## Methods

### Search strategy

To identify relevant available articles irrespective of language, the electronic databases of EMBASE, PubMed and the Cochrane Library were rigorously searched from inception to April 2020. The search terms included ‘urinary cancer’, ‘bladder cancer’, ‘kidney cancer’, ‘prostate cancer’ and ‘systemic immune-inflammation index’ or ‘SII’. Both MeSH terms and entry terms were utilized in the literature search. In addition, we screened all the references of the relevant studies and reviews to attain additional eligible studies.

### Inclusion and exclusion criteria

The studies included in the meta-analysis met the following inclusion criteria: (1) adult patients who were diagnosed with urinary cancer; (2) SII, which was defined as the multiplication of the neutrophil and platelet counts divided by the lymphocyte count, was available or could be calculated, and SII was presented as a binary variable with a selected cut-off value; (3) the primary outcome was overall survival (OS), and the relationship between OS and SII was analyzed; (4) the hazard ratios (HRs) with the 95% confidence intervals (95% CIs) were available or could be calculated; and (5) the study quality was assessed in accordance with the Newcastle–Ottawa quality assessment scale, and the included studies had a score of no less than 6.14 The exclusion criteria were as follows: (1) studies on the children or pregnant women; (2) experimental studies on the cell lines or animals; (3) the use of anti-inflammatory or immune-suppressive drugs in the studies; and (4) publication types including case reports, editorials, meta-analyses and reviews. When duplicated studies from the same population were included, the latest and most complete study was included.

### Data extraction

The following information was extracted from the selected studies: first author, publication year with the country or region of the study, study type, kind of cancer, number of samples, age of patients, follow-up time, cut-off value of SII and how the cut-off was selected, treatment that the patients received, stage of the cancer and data on the primary and secondary outcomes. Analysis results from univariate and multivariate analyses were extracted. Effect values in multivariate analysis were preferred, and subgroup analysis according to the different analysis methods was performed. If the HRs with the 95% CI were not available, they were calculated from survival curves using Engauge Digitizer. Two researchers extracted the information independently, and any disagreements were resolved by a third individual.

### Statistical analysis

The meta-analysis was performed using RevMan software (version 5.3; The Nordic Cochrane Center, Cochrane Collaboration, Copenhagen, Denmark). The HRs and 95% CIs from the survival analyses of the included studies were pooled to assess the prognostic role of SII in urinary cancer patients, and the odds ratios (ORs) with the corresponding CIs were pooled in the analysis of binary variables. The heterogeneity of the results across studies was qualitatively tested using Cochran’s Q-test and quantified using I2 statistics. I2 statistics of 25%, 50% and 75% represent the low, moderate and high levels of heterogeneity, respectively. A fixed-effects model was used when there was low heterogeneity; otherwise, a random-effects model was used. Publication bias was evaluated by funnel plots. Sensitivity analysis was performed by omitting individual studies one by one to assess the reliability of the results. A P value less than 0.05 was considered statistically significant.

## Results

### Search results and study characteristics

**Fig. 1:**
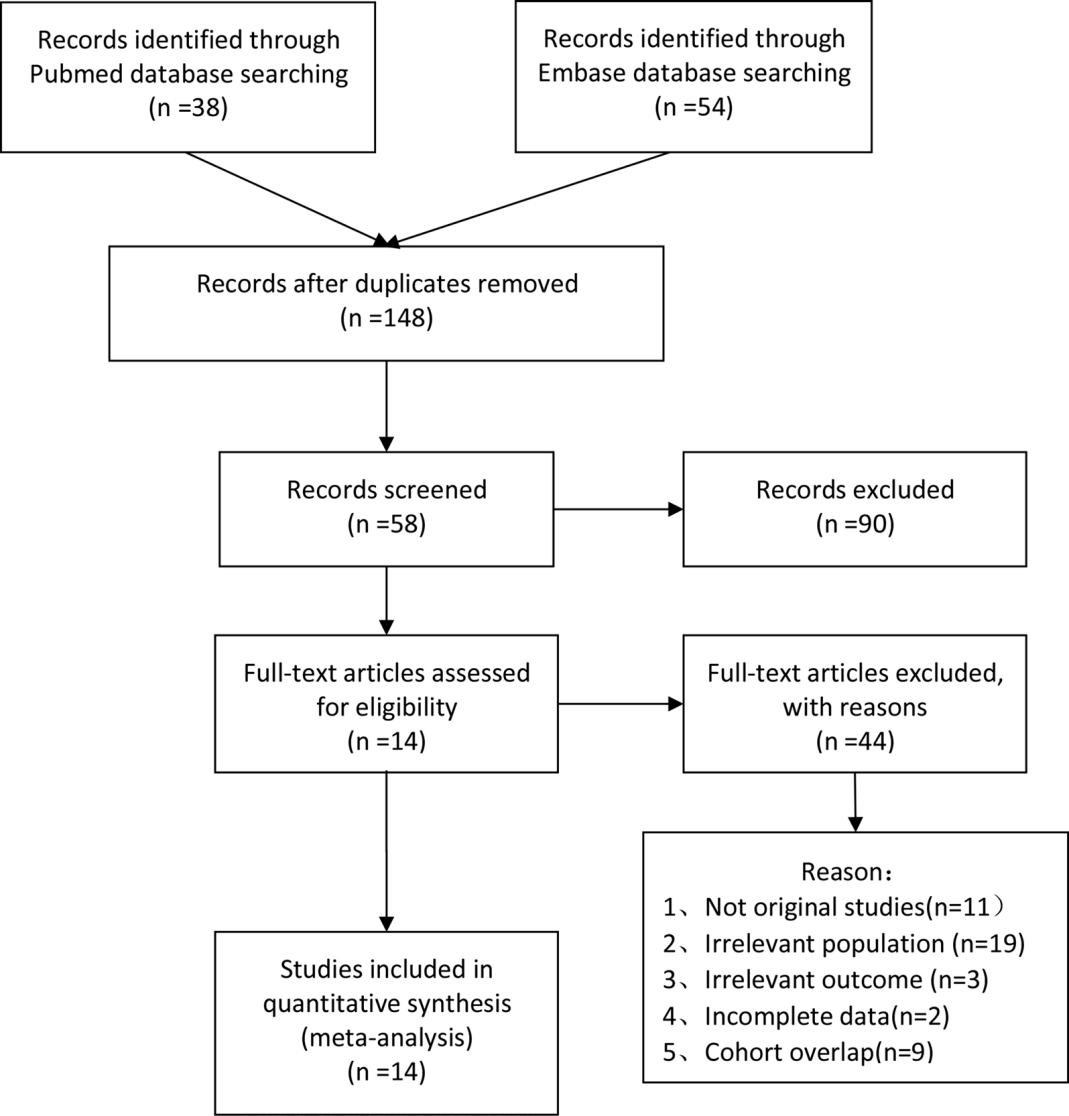
Flow chart for the study selection

The search yielded 184 studies, of which 76 studies were from the PubMed database and 108 studies were from the EMBASE database. No available studies were obtained from the Cochrane Library database. A total of 14 studies (11 full-text studies and 3 conference abstracts) were finally included in the present meta-analysis [9, 12, 13, 14–24]. Figure 1 shows the study selection process. There were 7 studies on patients with renal cancer, 5 of which were studies on advanced carcinoma, 1 on resectable carcinoma and the last one had unclear tumor stages. Among the studies on advanced renal cancer, the primary treatments were immunotherapy, targeted therapy and extensive surgeries. Three studies were on prostate cancer, and all of the included patients from these studies were diagnosed with metastasis. Abiraterone, docetaxel and their combination were selected as the first-line treatment in the three prostate cancer studies. Two studies evaluated the prognostic value of SII in patients with muscle-invasive bladder cancer after radical cystectomy. Two studies were conducted on patients with tumors from different organs. Several studies evaluated the prognostic role of other serum inflammation biomarkers. NLR (7 studies) and PLR (6 studies) were the most frequently studied biomarkers in previous studies. Three studies reported the association of the monocyte-to-lymphocyte ratio (MLR)/lymphocyte-to-monocyte ratio (LMR) and prognosis, and the prognostic role of the C-reactive protein-to-albumin ratio (CAR) was assessed in two studies. The details of the characteristics of the included studies are presented in Table 1.

**Table 1:** Characteristics of included studies

Study/year	Cancer type	Country/region	Study type	Included period	No of samples	Age	Primary outcome
Ugo De Giorgi (2019)	mRCC	Italy	P	2015–2016	313	65	OS/PFS
Cristian Lolli (2016)	mRCC	Italy	R	NA	335	63	OS/PFS
Wentao Zhang (2019)	BC	China	R	2015–2019	209	66.7	OS
Rebuzzi S.E. (2020)	mRCC	Italy	R	2016–2019	189	69	OS
Pawel Chrom (2018)	mRCC	Poland	R	2008–2016	502	NA	OS
Cristian Lolli (2016)	mCRPC	Italy	R	2011–2015	230	74	OS
Sacit Nuri Gorgel (2019)	MIBC	Turkey	R	2006–2018	191	62.1	OS/CSS
Ghanghoria A (2020)	RCC	India	R	NA	33	NA	OS
Hau-Chern Jan (2018)	UTUC	Taiwan	R	2007–2017	424	70	OS/PFS/CSS
Ya‑nan Man (2019)	mCRPC	China	R	2010–2018	179	70	OS
Emin Ozbek (2019)	RCC	Turkey	R	NA	176	62	OS/DSS
Liancheng Fan (2017)	mCRPC	China	R	2013–2017	104	72	OS/PFS
Palacka P (2017)	mUC	Slovakia	R	2000–2015	185	NA	OS/PFS
Sasanka Kumar Barua (2019)	mRCC	India	R	2012–2017	31	60	OS/PFS

Follow-up (months)	Cut-off	Cut-off selection	Treatment Methods	Stage/T stage	MVA	NOS score	Conference summary
24	1375	X-tile	Mix	T4	Y	7	N
49	730	X-tile	No surgery	T4	N	7	N
1–48	507	X-tile	Mix	Tis-T4	Y	6	N
NA	1375	NA	No surgery	T4	N	NA	Y
52.5	730	X-tile	Mix	T4	Y	8	N
1–30	535	X-tile	No surgery	T4	Y	7	N
37	843	ROC	Surgery	T2–T4	Y	8	N
6.8–38.6	8.67	NA	Surgery	NA	N	NA	Y
1–120	580	ROC	Surgery	Ta–T4	Y	8	N
24	535	NA	No surgery	T4	Y	7	N
NA	830/850	ROC	Surgery	T1–T4	N	7	N
1–50	200	ROC	No surgery	T4	Y	7	N
10	NA	NA	No surgery	T4	Y	NA	Y
NA	883	ROC	Surgery	T4	Y	6	N

mRCC: metastatic renal cell cancer; BC: bladder cancer; mCRPC: metastatic castration-resistant prostate cancer; MIBC: muscle invasive bladder cancer; RCC: renal cell cancer; UTUC: Upper-Tract Urothelial Carcinoma; mUC: metastatic urothelial carcinoma; P: prospective; R:retrospctive; OS: overall survival; PFS: progression-free survival; CSS: cancer-specific survival; DSS: disease-specific survival; MVA: multivariate analysis; NOS: Newcastle–Ottawa quality assessment scale; Y:yes; N: non

### Impact of SII on OS

**Fig. 2:**
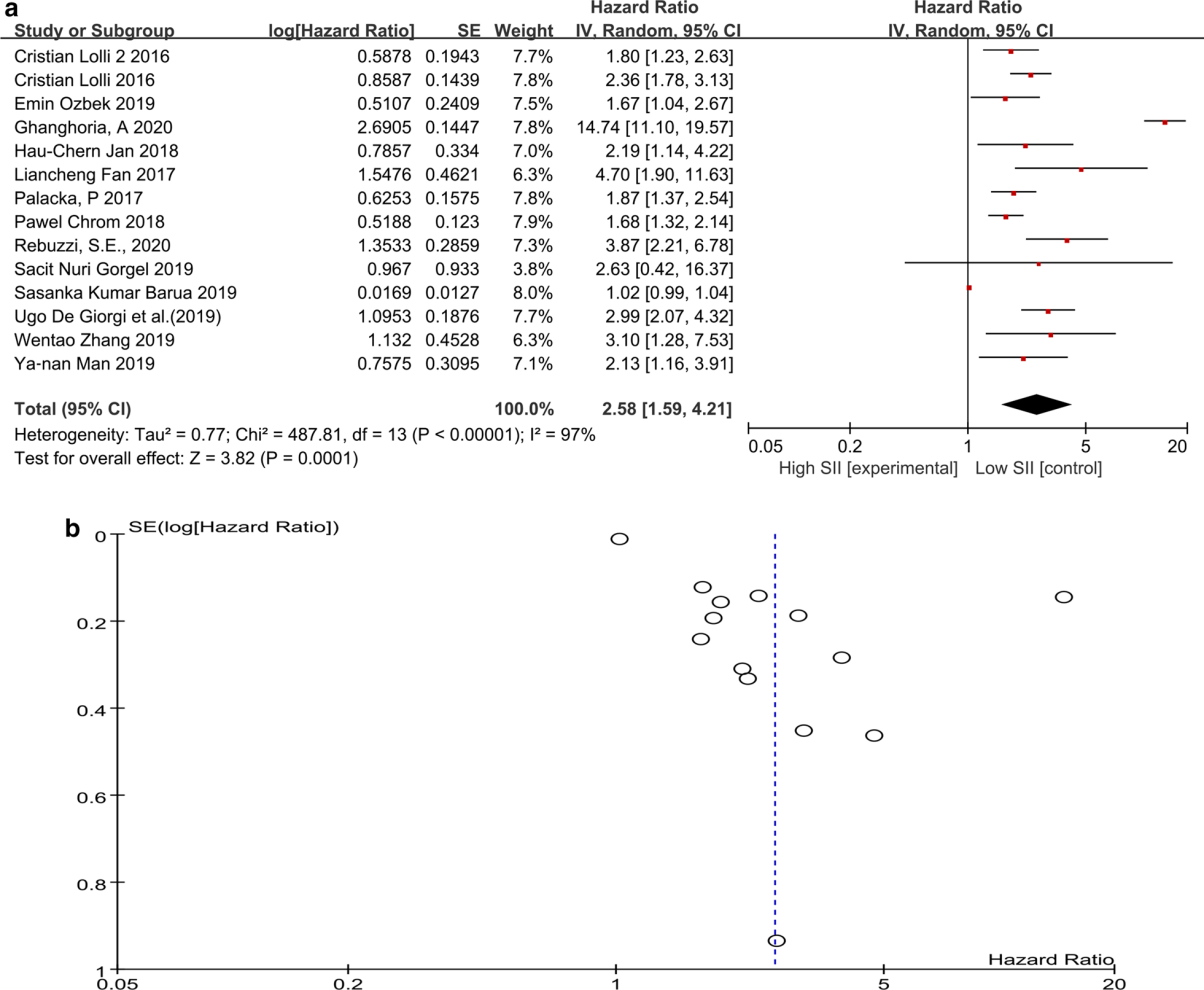
**a** Forest plot and **b** funnel plot of the overall survival in patients of high and low SII

The prognostic value of SII was evaluated in all 14 included studies. As shown in Fig. 2a, patients with high SII had a significantly better overall survival than patients with low SII (HR 2.58, 95% CI 1.59–4.21, p = 0.0001). High heterogeneity was observed; therefore, a random-effects model was used in the analysis. There was no significant publication bias, as shown in the funnel plot (Fig. 2b). Then, we performed the subgroup analysis (Table 2). The subgroup analysis according to the cancer type, study type, cut-off value of SII and analysis method showed that the poorer prognosis was persistent in patients with high SII than in patients with low SII. All the above analysis results were evaluated to be reliable after the sensitivity analysis.

**Table 2:** Results of subgroup analysis of pooled hazard ratios of OS of patients with different SII

Stratified analysis	No. of studies	Pooled HR (95% CI)	P‐value	Heterogeneity	
				I^2^ (%)	P_Q_
Cancer type					
RCC	7	2.73 (1.28, 5.81)	0.009	99	< 0.001
PC	3	2.29 (1.44, 3.63)	< 0.001	46	0.160
BC	2	3.01 (1.35, 6.68)	0.007	0	0.870
Unclear	2	1.92 (1.46, 2.54)	< 0.001	0	0.660
Urothelial cancer	4	2.03 (1.55, 2.63)	< 0.001	0	0.730
Non urothelial cancer	10	2.67 (1.46, 4.88)	0.001	98	< 0.001
Study type					
Prospective	1	2.99 (2.07, 4.32)	< 0.001	NA	NA
Retrospective	13	2.55 (1.53, 4.27)	< 0.001	97	< 0.001
Treatment					
Surgery	5	1.70 (1.04, 2.79)	0.030	76	0.002
Non surgery	7	2.16 (1.74, 2.68)	< 0.001	52	0.050
Mix	2	6.67 (1.40, 31.84)	0.020	98	< 0.001
Cut-off value					
0–500	2	9.05 (2.99, 27.41)	< 0.001	82	0.020
501–1000	9	1.84 (1.30, 2.59)	< 0.001	90	< 0.001
>1000	2	3.23 (2.38, 4.39)	< 0.001	0	0.450
Analysis					
Multivariate	10	2.06 (1.44, 2.94)	< 0.001	9.1	< 0.001
Univariate	1	2.36 (1.78, 3.13)	< 0.001	NA	NA

CI: confidence interval; HR: hazard ratio; OS: overall survival; SII: systemic immune‐inflammation index

### Impact of SII on progression‐free survival (PFS)

We performed the analysis with PFS as the secondary outcome. As shown in Table 3, patients with high SII had a worse prognosis than patients with low SII (HR 1.92, 95% CI 1.29–2.88, p = 0.001). Subsequently, subgroup analysis was performed according to the cancer type, treatment type, study type and analysis method (Table 4). A significant difference between patients with high and low SII in terms of PFS was observed in almost all the subgroup analyses, except for the analysis in prospective studies or in patients who underwent surgery.

**Table 3:** Analyses of secondary outcomes in urologic cancers

Secondary outcomes	No. of studies	No. of cases	Pooled HR (95% CI)	P-value	Heterogeneity	
					I^2^	Model
PFS	7	1554	1.92 (1.29, 2.88)	0.001	93	Random
CSS	2	600	2.58 (1.36, 4.91)	0.004	7	Random
ORR	2	448	0.40 (0.22, 0.71)	0.002	0	Fixed
DCR	2	448	0.93 (0.11, 8.05)	0.950	95	Random

CI: confidence interval; CSS: cancer‐specific survival; DSS: disease‐specific survival; ORR: overall response rate; DCR: disease control rate; HR: hazard ratio; SII: systemic immune‐inflammation index

**Table 4:** Results of subgroup analysis of pooled hazard ratios of PFS of patients

Stratified analysis	No. of studies	Pooled HR (95% CI)	P‐value	Heterogeneity	
				I^2^ (%)	P_Q_
Cancer type					
RCC	4	1.52 (0.99, 2.31)	0.050	91	< 0.001
PC	1	11.8 (5.6, 24.87)	< 0.001	NA	NA
Unclear	2	1.6 (1.23, 2.07)	< 0.001	0	0.900
Urothelial cancer	2	1.6 (1.23, 2.07)	< 0.001	0	0.900
Non urothelial cancer	5	2.31 (1.23, 3.69)	0.007	95	< 0.001
Treatment					
Surgery	2	1.16 (0.8, 1.7)	0.430	62	0.110
Non surgery	4	2.65 (1.53, 4.58)	< 0.001	88	< 0.001
Mix	1	1.43 (0.89, 2.3)	0.140	NA	NA
Study type					
Prospective	1	1.43 (0.89, 2.3)	0.140	NA	NA
Retrospective	6	2.03 (1.29, 3.2)	0.002	94	< 0.001
Analysis					
Multivariate	5	1.93 (1.14, 3.29)	0.010	93	< 0.001
Univariate	2	1.91 (1.53, 2.38)	< 0.001	0	0.550

CI: confidence interval; HR: hazard ratio; OS: overall survival; SII: systemic immune‐inflammation index

### Impact of SII on cancer-specific survival (CSS), overall response rate (ORR) and disease control rate (DCR)

As shown in Table 3, patients with low SII had a significantly better cancer-specific survival than patients with high SII (HR 2.58, 95% CI 1.36–4.91, p = 0.004). Low SII was evaluated to be associated with a higher ORR (OR 0.40, 95% CI 0.22–0.71, p = 0.002). However, the difference in terms of DCR was not significant between patients with high and low SII (OR 0.93, 95% CI 0.11–8.05, p = 0.950).

## Discussion

To our knowledge, this is the first meta-analysis that analyzed the prognostic value of SII in urologic cancers. A total of 14 published articles or conferences with 3074 cases were included in this study. From the pooled results, we found that UC patients with a high SII value had a worse prognosis for OS (HR 2.58, 95% CI 1.59–4.21). Moreover, we performed subgroup analysis to assess the prognostic significance of SII. The subgroup analysis results showed that high SII was a prognostic marker for worse OS in PC (prostate cancer) and UC (urothelial carcinoma). Similarly, high SII was also negatively correlated with PFS, CSS, and ORR. Considering the above results, SII could serve as a prognostic factor for urinary cancers.

Currently, an increasing number of biological markers have been applied in clinical work due to their inexpensiveness and ready availability. The lymphocyte count, plasma fibrinogen, NLR, PLR and LMR have been proven to be valuable for the prognosis of cancer patients. However, when only one or two parameters were involved, these predictors became unstable and tended to be susceptible to the influence of other confounding factors [25]. SII, defined as P (platelet count) x N (neutrophil count)/L (lymphocyte count), combines NLR with platelet count and might have a better predictive power than NLR [26]. As a more objective tumor marker, SII reflects the balance between host inflammation and the state of the immune response [27].

SII has been reported in other studies as a predictor for cancer outcomes, such as small cell lung cancer, GI (gastrointestinal) cancer, and hepatocellular carcinoma [7, 8, 25]. The prognostic role of SII in tumors can be explained by the following mechanisms. Numerous studies have reported the relationship between inflammation and cancer and found that cancer-related inflammation is an indispensable component of the tumor microenvironment [28, 29]. Circulating inflammatory cells, such as neutrophils, lymphocytes, and platelets, play important roles in the development and progression of tumors [5, 33]. Patients with cancer often suffer from a hypercoagulable state, and platelets can mediate the survival and growth of tumor cells by regulating the formation of micrometastases [30]. Lymphocytes inhibit the proliferation and growth of tumor cells by cytotoxic cell death in cancer immune surveillance and resistance [31, 32]. In addition, neutrophils play an important role in metastasis and progression [5, 33]. Thus, SII could explain why higher levels of neutrophils and platelets and lower levels of lymphocytes indicate a weak immune response but a strong inflammatory response.

The limitations of this study include the following aspects. First, most of the articles included in this study were retrospective studies, and only one was a prospective study. Second, the number of studies that met the requirements was not that large, and the sample size included was relatively small, especially in the subgroup analysis. Third, the cut-off values of SII varied in different studies, and the calculation methods were inconsistent. A few studies did not provide multivariate analysis results, so we used univariate results instead. Finally, despite the subgroup analysis and the sensitivity analysis being performed, we were not able to confirm whether different types of tumors and different treatments would lead to bias in the results.

In conclusion, the outcomes presented in this meta-analysis indicated that high SII was independently related to poor prognosis in patients with urologic cancers. SII could be a significant and cost-effective prognostic indicator for urinary cancers. Of course, well-designed, large-scale multicenter studies are needed to validate the clinical value of SII as a prognostic biomarker for urologic cancers.

## Data Availability

The datasets used in this study are available from the corresponding author upon reasonable request.
